# The Interplay Between Helping Behavior and Absenteeism in Teams: A Longitudinal Examination of Their Reciprocal Relationship in a Public Organization

**DOI:** 10.1177/00910260241226947

**Published:** 2024-01-24

**Authors:** Michel Tremblay

**Affiliations:** 1 HEC Montréal, Québec, Canada

**Keywords:** within-team variability, behavior change, OCB-helping, absenteeism

## Abstract

This study employed a longitudinal model to investigate the reciprocal relationships between interpersonal citizenship behavior (OCB-I) and absenteeism at the team level. The research utilized four waves of data from a sample comprising over 5,000 employees in 168 teams within a large Canadian public organization. Drawing upon the focus theory of normative conduct and the collective identity perspective, our findings indicated that a positive change in OCB-I, which encompasses helping behaviors, led to a subsequent decrease in team absenteeism. In addition, emphasizing the identity perspective and allocation of time perspective, our study demonstrated that increased absenteeism within a given period was associated with a subsequent reduction in team OCB-I.

In today’s landscape, public organizations are encountering heightened scrutiny and escalating performance expectations from both citizens and political representatives, all the while grappling with the challenge of preserving service levels amid diminishing budgets (De Geus et al., 2020; Vigoda-Gadot & Golembiewski, 2005). In the pursuit of cost-effective solutions and enhanced productivity, it becomes imperative to adopt strategies that ensure the ongoing delivery of excellent services and maintain high levels of satisfaction among the public stakeholders.

Evidence show from a number of countries that short- and long-term absenteeism are higher in the public sector than in the private sector (Hansen et al., 2019; Mastekaasa, 2020). A survey has indicated that, on average, supervisors were perceived to be 15% less productive, while coworkers were considered to be 29.5% less productive when covering for a typical absence day (in Koumenta, 2015). Absenteeism, as a significant cost factor, has led to a growing focus on reducing it in numerous public human resource departments. This issue has also garnered increasing attention within the public literature, as evidenced by recent research studies (e.g., Jensen et al., 2019; Løkke & Krøtel, 2020; Mastekaasa, 2020).

However, public administration scholars have begun to underscore the importance of organizational citizenship behavior (OCB) in public organizations. OCB is broadly defined as behavior that is discretionary by not formally rewarded by the organization but it improves the functioning of organization (Organ, 1988). Findings in public and private sectors have encouraged public organizations to use citizenship behaviors to increase organizational performance (Vigoda-Gadot & Golembiewski, 2005). Preliminary evidence suggests that public sector employees tend to exhibit a higher inclination toward people-oriented and altruistic citizenship behaviors (OCB-I) when compared with their private sector counterparts, whereas private sector employees are more likely to engage in citizenship behavior directed toward the organization (OCB-O, Glińska-Neweś & Szostek, 2018; Grego-Planer, 2019). An underexplored research question in the public sector pertains to the potential interplay between short-term absenteeism within public teams and the cultivation of OCBs. Conversely, it remains to be investigated whether fostering greater citizenship behaviors within public teams might serve as a partial solution to address the issue of absenteeism in the public sector.

Recent literature reviews on OCB and absenteeism in the public sector (e.g., De Geus et al., 2020; Mastekaasa, 2020) indicate that several research gaps were uncovered. One notable gap is that the majority of studies on OCB have predominantly focused on the private sector. Investigating more specifically how OCBs may contribute to reducing absenteeism in public teams carries both theoretical and practical significance. From a theoretical perspective, such research may shed light on the ongoing debate surrounding the impact of OCB on counterproductive or deviant behavior (CWB), as discussed by Spector and Fox (2010). Exploring the relationship between OCB and absenteeism, widely viewed as a mildly deviant behavior (Johns & Miraglia, 2015), can contribute to a deeper understanding of how public employees’ behaviors are interrelated. This knowledge may help to build a more comprehensive and nuance theories of workplace behavior. In practical terms, according to Vigoda-Gadot and Cohen (2004), citizenship behavior holds significant importance for the effective functioning of public systems and administrative bureaucracy. Enhanced levels of OCB within the public sector hold the potential to markedly enhance the quality and efficiency of public services, ultimately contributing to the establishment of a positive and esteemed public sector image. Consequently, this may bolster citizen trust and confidence in the delivery of public services. A Canadian study revealed that a 1% increase in absenteeism was related to a .44% decrease in productivity (Zhang et al., 2017). Given the negative impact of workplace deviance behavior on business performance (Dunlop & Lee, 2004), understanding whether encouraging team OCB in public sector may prevent voluntary absenteeism, this knowledge can be used to design interventions and strategies to promote OCB to mitigate absenteeism.

Second, studies into the antecedents of OCB and absenteeism have predominantly focused on the individual-centered characteristics, dispositions, job attitudes, and motivations. Over the last few decades, researchers have also delved into the impact of social context on such team behaviors (e.g., Ehrhart & Naumann, 2004; Miraglia & Johns, 2021). In today’s public sector, the increasing significance of teams as a managerial unit is driven by the demand of more adaptable, agile, and responsive governance structures (Ali et al., 2021; Mergel et al., 2021). Examining how positive team behaviors, as team helping, can act as preventive measures against the development of a short-term absenteeism culture is highly relevant. Addressing the group-level perspective can provide valuable insights into the dynamics of OCB and absenteeism and guide more effective public interventions and policies.

Third, there has been a notable absence of research within the public sector that delves into the intricate OCB-absenteeism relationship. To the best of our knowledge, existing research has primarily concentrated on exploring the impact of team OCB on absenteeism (e.g., N. P. Podsakoff et al., 2009; Whitman et al., 2010). However, it often remains unclear whether absenteeism at team level acts a precursor to other outcomes or behaviors (McGrandle & Ohemeng, 2017). Enhancing our comprehension of the barriers that curtail the benefits associated with OCB in public sector hold significance because it is widely acknowledged that such behavior is a key driver of group effectiveness and success (P. M. Podsakoff et al., 2018). Therefore, gaining insights into whether absenteeism may hinder organizational endeavors to cultivate a pervasive culture of helping and social support holds notable practical significance for public managers. Second, by examining change rather only levels, researchers can establish causality and better understand the direction of influence between OCB and absenteeism. It helps answer compelling or contradictory research questions as:

Research Question (RQ1): Does a change in OCB precede and cause changes in absenteeism, or is it the other way around?

By understanding the direction of influence, researchers can provide more accurate and meaningful insights. A dynamic perspective acknowledges that workplace behaviors, such as OCB and absenteeism, are not static but subject to change. Theoretical frameworks that consider change offer a more realistic representation of how these behaviors interact in the evolving work environment. This dynamic view aligns with the complexity of human behavior and organizational dynamics. Theoretical insights into changes can inform the development of effective interventions and policies. If the research identifies a causal relationship, organizations can design targeted strategies to promote beneficial changes in OCB and reduce absenteeism.

We employ a comprehensive theoretical framework, integrating the social information processing theory (SIP, Salancik & Pfeffer, 1978), the focus theory of normative conduct (FTNC; Cialdini & Trost, 1998), and social identity theory (SIT; Tajfel & Turner, 1979), to elucidate the reciprocal relationship between OCB and absenteeism at team level. Our study is based on a longitudinal research design, encompassing four-wave longitudinal data from over 5,000 employees organized into 168 teams within a prominent public organization in Canada, allowing us to rigorously test our hypotheses.

## Theoretical Foundations

We draw on SIP (Salancik & Pfeffer, 1978) to better understand the emergence of shared conducts or behaviors in work teams, and on the FTNC (Cialdini & Trost, 1998) to identify the normative forces and pressures explaining why team members are more likely to align their behaviors to those of their relevant environment. SIP theory holds that employees’ perceptions of their environment are shaped to a large part by their social environment. When individuals lack readily available, concrete data about their work environment, they often turn to social information obtained through observations, comments or discussions with colleagues to shape their job-related attitudes and behaviors. We contend that team members use the growth or decline of helping and absenteeism within their work team to establish norms of conduct and guide their behavior.

The influence of group behavior depends on whether norms are descriptive or injunctive (Cialdini et al., 1991). Descriptive norms develop from observing what other team members do in certain situations. The more a group adopts the same behavior in a given occasion, the more the group members will tend to see this behavior as appropriate or morally legitimate. Injunctive norms, unlike prescriptive norms, develop through normative influence, or when group members feel pressure to perform an appropriate, as well as inappropriate behavior (Cialdini & Trost, 1998). There is a growing evidence that social norms play a significant role in individual OCB and CWB display (Jacobson et al., 2015, 2020; Meyer et al., 2010). Although the two aforementioned theories are valuable in elucidating why employees engage in distinct behaviors such as OCB or CWB, these theoretical frameworks offer an incomplete explanation for how a shift in a normative behavior (e.g., OCB-I) might influence a corresponding change in another normative behavior (CWB). To address this gap, we integrate SIT (Tajfel & Turner, 1979) to shed light on the motivation driving team members to engage in consistent and congruent behaviors.

SIT suggests that people categorize themselves and others into social groups based on shared characteristics, such as gender, ethnicity, profession, or even sports team allegiance. These group affiliations become an essential part of an individual’s self-concept, contributing to their sense of identity. Self-identity serves both to differentiate oneself from others and to conform to the values, beliefs, and behaviors of the social groups to which one belongs. Collective self-identity is the part of an individual’s self-concept that is derived from their perceived membership in various social groups or categories. It involves the sense of belonging and identification with specific groups and the incorporation of those group memberships into one’s self-definition. The salience of a particular identity can vary depending on the social context and collective self-identity can influence group dynamics, as individual conform to group norms and values and support to the group’s goals and interests (Jackson et al., 2006; Tajfel & Turner, 1979). A given self-definition is adopted or become more salient at the moment at which the context-dependent identity becomes activated. Based on above theoretical perspectives, we posit that as a particular behavior (OCB or absenteeism) becomes increasingly expected or normative within a group, team members are more inclined to embrace compatible behaviors to uphold and reinforce their positive self-identity.

### Temporal Relationship Between OCB-I and Absenteeism to the Team Level

This study centers its attention on OCB directed at individuals (OCB-I), often associated with affiliative or interpersonal forms of OCB. As reported by N. P. Podsakoff et al. (2014), a substantial portion—75%—of OCB studies predominantly examine these behaviors, which frequently involve assisting colleagues with heavy load or work-related challenges (Ehrhart, 2018). Furthermore, empirical evidence supports the notion that OCB-I is more commonly observed within the public sector as compared with the private sector, as indicated by studies such as Grego-Planer (2019) and Glińska-Neweś and Szostek (2018). This observation underscores the significance of this particular form of OCB in public sector contexts. In the current research, we focus on a specific facet of CWB-absenteeism. Absenteeism is characterized as the failure to report to work as scheduled, as defined by Johns (2002). This study delves into the influence of time lost (total days of absence) and frequency of absenteeism. The current study emphasizes a focus on short-term absenteeism rather than long-term absenteeism. This choice is based on the assumption that short-term absenteeism exhibits a stronger association with motivation when compared with long-term absenteeism. It is worth highlighting that, in contrast to long-term absenteeism, short-term absenteeism generally does not necessitate a physician’s certification. This aligns with research findings indicating that motivational factors predominantly influence short-term absenteeism (Ferrie et al., 2009).

We posit that influence of higher OCB-I on absenteeism may occur through two forces or pathways: 1—on voluntary basis through the increase of citizenship identity; 2—on involuntary basis through the pressure to avoid negative consequences as exclusion. First, we suggest that a higher descriptive norm of OCB in teams than was usual would elicit a higher citizenship identity among team members (Karolewski, 2009), leading to a decrease in absenteeism. As categorization of self as member of social group depends on range and the significance of stimuli, a higher OCB-I would elicit a higher collective citizenship identity because a greater focus is centered on “us,” rather than “I.” When more team members engage in OCB-I than in the past, more members experience a sense of pride and self-identity associated with OCB-I. OCB-I reflects behaviors that are supportive, helpful, and cooperative among team members. As these behaviors become increasingly common and expected within the team, they contribute to the development of positive behavioral norms. These norms promote a sense of unity and shared identity within the group, as team members recognize and adhere to these established standards. A higher citizenship identity within the team suggests the development of greater responsibility and reliability norms. Team members who share this identity are more inclined to follow these norms, which discourage absenteeism. A strong citizenship identity involves a deep commitment to the values and principles associated with being a responsible and supportive team member. Team members who strongly identify with their role as a good citizen within the team are more likely to uphold these values, which include a commitment to attendance. Furthermore, team members who strongly identify with the group are motivated to demonstrate loyalty to their colleagues by attending work as scheduled, contributing to a low level of absenteeism. They are also more likely understand that their attendance or absenteeism has an impact on the team’s overall performance. This sense of collective accountability acts as a deterrent to absenteeism. Greater OCB-I than normally involves that increasing team members experience social support, a key resource that may reduce absenteeism by offsetting the negative effects of higher job demands and strengthening various motivational states as job and team commitment (Miraglia & Johns, 2021).

Second, a positive change in OCB-I in a team also may reduce absenteeism rate through it impact of injunctive pressure because the cost to engagement in CWB when OCB-I in team increase may result in harmful personal outcomes as exclusion or ostracism. The social influence literature suggests that people are motivated to avoid deviance from their normative in-group’s positions (Turner, 1991). When a higher proportion of team members help others, the situation becomes stronger. Meyer et al. (2014) have provided evidence that one important indicator of situation strength was consequences to deviate from what is expected. Consequently, social pressure to perform compatible behavior is more likely to be salient when OCB-I increases because performing deviant behavior is more likely to lead to severe consequences as the reduction of support, and exclusion of the group. Team members often are aware that their behavior is subject to social scrutiny, and they are more likely to conform to the group’s expectations to avoid social disapproval or potential criticism. Bolino et al., (2010) have introduced the notion of citizenship pressure, which they defined “as a specific demand in which employees feel pressured to engage in OCBs” (p. 236). This citizenship pressure can even motivate or force individuals to come to work even if they are ill. Emerging research has started to link presenteeism with OCB (Miraglia & Johns, 2016). Presenteeism is defined as the act of attending work despite being in ill health (Johns, 2010). A heightened norm of OCB-I is more likely to generate increased expectations for citizenship behaviors (Bolino et al., 2010), encouraging attendance at work through the phenomenon of presenteeism. A recent study by Boekhorst and Halinski (2023) has revealed that coworker presenteeism was found to be indirectly associated with OCBs through the mechanism of citizenship pressure. Although their study’s focus differs from ours, this research suggests that when OCB-I becomes a stronger norm within a given context, the pressure to engage in such extra-role behavior increases for each team member, compelling them to come to work to even when unwell to avoid sanctions, reducing absenteeism.

The negative relationships between OCB and CWB and OCB and absenteeism have received broad support either at the individual level or group level, or at within-person (e.g., Dalal, 2005; MacKenzie et al., 2018; N. P. Podsakoff et al., 2009), however, examination of this relationship at within-team level in the public sector has been overlooked. Therefore, we propose the following hypothesis:

**Hypothesis 1 (H1):** Temporal increase (decrease) in team OCB-I is related to temporal decrease (increase) in team absenteeism.

### Temporal Relationship Between Absenteeism and OCB-I to the Team Level

An underexplored research question pertains to the impact of absenteeism on citizenship behavior within public teams. We sustain that team absenteeism may affect helping behavior through two different pathways; 1—via normative expectations and social identity and 2—via workload. As defined above, workplace norms are shared expectations about how employees should behave and interact with each other (Salancik & Pfeffer, 1978). In a workplace where absenteeism is common and tolerated, it sends a signal to employees that such behavior is acceptable or even expected. This leads to the emergence of an absenteeism culture generally described as to “the set of shared understandings about absence legitimacy in a given organization and the established ‘custom and practice’ of employee absence behavior and its control” (Johns & Nicholson, 1982, p. 136). When norms of absence are elevated in a given time, this higher legitimacy of absences shapes the group identity with various labels that may include “Absence-Heavy Team,” ’Take-off Days Team or “Deviant Team.” When absences become more and more normative and acceptable, individuals are more likely to adjust their own behavior to conform to these norms. They may assume that prioritizing personal commitments over work responsibilities, including helping coworkers, is a legitimate behavior. In addition, there is a proposition that workplace norms often possess a self-reinforcing nature, as suggested by Cialdini (2007). When absenteeism becomes a norm and highly cultural, it can be challenging to break that cycle and establish new norms that promote responsibility and helping behavior. Those who try to challenge existing norms may face resistance or even backlash from colleagues who are comfortable with the status quo. Furthermore, SIT posits that individual behavior is influenced by the desire to maintain a positive social identity. In a workplace with a higher culture of absenteeism, individuals may identify more with the “absenteeism group” and less with the “committed and responsible group.” As a result, they may be less motivated to engage in helping behavior that is seen as normative in the “committed” group. When absenteeism becomes pervasive, team members may reduce their helping behaviors to align their conduct with the group’s perceived norm, even if the norm is less supportive or helpful due to the emergence of stronger absenteeism culture. We cannot rule out the possibility that an increase in absenteeism poses a threat to the team’s identity, especially if it diminishes cohesion and fosters conflicts. In response to this potential threat, team members may become less motivated to offer assistance due to uncertainties about whether their help will be reciprocated in the future.

We also posit that absences compel team members to allocate time and resources differently, resulting in an increased of workload and decreased energy levels. According to time allocation theory (Bergeron, 2007; Nielsen et al., 2012), the fixed amount of time devoted to prescribed team activities must be reallocated among other team members; thus, a choice must be made between task performance and OCB-I. Consequently, team members must perform more in-role backup behaviors to ensure the team reaches its objectives (Barnes et al., 2008). These behaviors occur when peers are unable to do so themselves. Moreover, increasing in-role backup behaviors becomes taxing when absenteeism is higher than normally. Most team members experience increased workload that consumes valuable resources such as energy (Duff et al., 2015). Furthermore, when an increasing proportion of employees in a unit are absent, the remaining team members feel the pressure to increase their efforts to ensure the team fulfills its task obligations. The higher the level of absenteeism in each period, the more in-role backup behaviors team members must perform to ensure the team reaches its objectives (Barnes et al., 2008). This extra effort comes at a cost, namely the risk of significantly depleting collective energy, preventing teams from engaging in teamwork activities such as OCB-I. Hence, we propose the following hypothesis:

**Hypothesis 2 (H2):** Temporal increase (decrease) in team absenteeism is related to temporal decrease (increase) in team OCB-I.

## Method

### Sample and Procedure

The data for this study were provided by a large Canadian organization in the public sector. This organization is government-owned electric utility, and one of the largest and most prominent electricity producers and distributors in North America. This public organization is actively involved in research and development in field of energy. We used longitudinal data collected over 4 consecutive years (four measurement intervals) to test the change hypothesis. The level of analysis is the team and each employee belongs to a team. A team is part of a group that reports to a supervisor or formal leader. The majority of these teams work in project structures. The performance goals and reward systems of this public organization are primarily collective rather than individual, such that the level of interdependence of these team members is relatively high (Courtright et al., 2015). For the purposes of this study, the teams must meet the following criteria: (a) comprise full-time employees; (b) remain complete or operational during the 4 years covered by the survey; (c) include at least three respondents; (d) have no change of immediate superior during the study period; and (e) team absenteeism data must be collected during the same period. Table 1 summarizes survey sampling statistics for each period.

**Table 1.** Survey Sampling and Work-Unit Consensus Statistics.

Year of survey administration				
Statistics	T1	T2	T3	T4
Number of potential respondents	7,659	8,508	9,616	9,003
Number of completed surveys	4,519	4,935	5,770	5,312
Return rate	57%	58%	60%	59%
Average number of respondents per unit	26.90	29.37	34.34	31.62
Number of work units	168	168	168	168
Range of employees per unit	3–137	3–144	3–184	3–154
Average R_wg_ for OCB-I	.85	.86	.87	.87
Average A_wg_ for OCB-I	.67	.68	.68	.67
ICC(1) for OCB-I	.07	.05	.04	.04
ICC(2) for OCB-I	.67	.60	.61	.55

*Note.* OCB = organizational citizenship behavior.

### Measures

The survey was conducted by an independent private consulting firm and the organization under assessment. The questionnaire items were adapted to this organization’s work context and were negotiated with key stakeholders (e.g., unions).

#### OCB-Helping

OCB-helping was assessed using three indicators (degree of helping within the team, degree of cooperation between team members, and degree of cooperation with other unit team members) on a 10-point Likert-type scale varying from “*very low*” (1) to “*very high*” (10). This scale is consistent with interpersonal facilitation dimension of contextual performance construct (Van Scotter & Motowidlo, 1996), and previous group OCB studies (e.g., X.-P. Chen et al., 2005; Choi & Sy, 2010). The alpha coefficient for the OCB-helping construct was .81. Responses were aggregated at the unit level by averaging individual-level responses for each unit. According to G. Chen et al. (2004), researchers must question the level of agreement between the respondents of each unit and evaluate the degree of variation among units. First, we used rwg and awg to assess within-team agreement (James et al., 1984). Table 1 shows that average rwg values for OCB-I in each period were .85, indicating an acceptable level of agreement. This level of agreement is comparable to previous studies (e.g., Ehrhart et al., 2006). Although rwg values have been widely used to compute within-group agreement, they have several limitations, such as dependency on a scale and sample size and bias caused by the uniform null distribution assumption (Brown & Hauenstein, 2005). An alternative approach for computing within-group agreement is awg (j) (Brown & Hauenstein, 2005). Table 1 shows that average awg values for OCB-I in each period was .67, indicating a moderate level of agreement according to the guidelines outlined by LeBreton and Senter (2008). We also estimated ICC (1), defined as the ratio of between-group variance, and ICC (2), defined as the level of reliability of group means using individual-level data (Lebreton & Senter, 2008). Over the four periods studied, the average ICC (1) for OCB-I was .05 and .60 for ICC (2). These intra-class correlation coefficients are comparable to previous studies (e.g., Hausknecht et al., 2008) and suggest a medium effect of group membership, supporting OCB-I aggregation at the team level.

#### Absenteeism

Contrary to previous studies, this study used a specific objective CWB (absenteeism) rather than a subjective global or specific self-report CWB measure (e.g., self-rating of using sick leave when not sick). Consistent with previous research (e.g., Dineen et al., 2007), absenteeism was conceptualized as an additive model and thus has been operationalized at the team level as the mean level within a team. Absenteeism data were provided by the organization and referred to work absence episodes of 5 days or less. Thus, this study focused mainly on short-term absenteeism. Two absenteeism measurement indexes are commonly used, duration (*“time lost index”*) and frequency (*“frequency index”*). These represent the sum of time away from work and the number of absence episodes over a specific period, respectively (Steel, 2003). Absences were calculated at the end of the year for each individual. We calculated the average frequency of absences and days lost within each unit consistent with past research (Diestel et al., 2014; Hausknecht et al., 2008).

#### Control Variables

We controlled for unit size, the proportion of males, average salary, average age, average seniority, and job satisfaction to mitigate concerns of misspecifications. These are predictors for OCB or unit-level absenteeism (Dello Russo et al., 2013; Whitman et al., 2010). The age and sex of participants are frequently controlled demographic variables when researchers study absenteeism on an individual level (see Johns, 1997). Moreover, seniority proved to be a particularly relevant variable, given that in the assessed organization, the number of days of absence granted is based on seniority. Job satisfaction has been theorized as an alternative explanation to positive or negative behaviors (see Dalal et al., 2009; Dineen et al., 2007). Findings show a negative relationship between satisfaction and absenteeism (Miraglia & Johns, 2016), that job satisfaction is more strongly related to short than long-term absenteeism (Hassan et al., 2014), and that satisfaction is positively related to OCB (Whitman et al., 2010). We controlled for various job satisfaction dimensions (e.g., superior, organization, colleagues, empowerment, justice, recognition) with 15 items assessed on a 10-point Likert-type scale varying from “*not satisfied at all*” to “*extremely satisfied*.” The alpha coefficient for the overall job satisfaction measure was .93. Responses were aggregated at the unit level by averaging the individual-level responses for each unit. Over the four periods studied, the average ICC (1) for job satisfaction was .08 and .71 for ICC (2).

### Analysis

**Figure 1.**
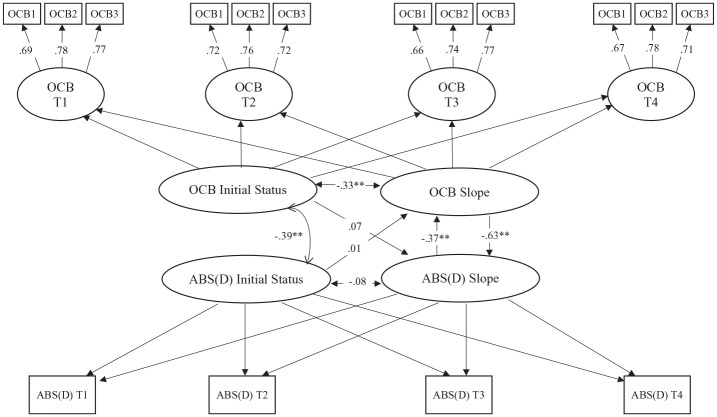
LGM Results for Absenteeism Duration (Team-Level). *Note. Model fit*: x2 = 47.24** (24); RMSEA = .047; CFI = .969; TLI = .964. LGM = latent growth model; RMSEA = root mean square error of approximation; CFI = comparative fit index; TLI = Tucker–Lewis Index.

**Figure 2.**
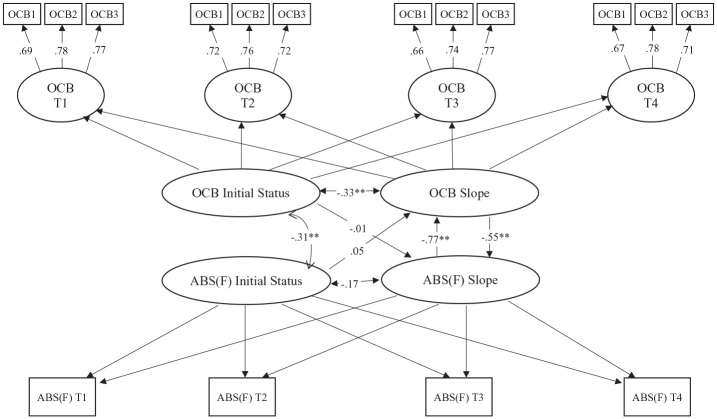
LGM Results for Absenteeism Frequency (Team-Level). *Note. Model fit*: x2 = 43.76** (24); RMSEA = .045; CFI = .972; TLI = .966. LGM = latent growth model; RMSEA = root mean square error of approximation; CFI = comparative fit index; TLI = Tucker–Lewis Index.

We used the latent growth model (LGM) and structural equation model with MPLUS 6.0 (Muthén & Muthén, 1998–2010) to examine whether changes in OCB lead to changes in absenteeism over time and vice versa. The LGM is flexible and precise to model time in longitudinal research (Howardson et al., 2017). The fundamental difference between traditional longitudinal techniques (e.g., lagged regression or use of change scores) and the LGM approach is that the latter captures change as a latent variable and examines the variance between individuals or teams (Maia et al., 2016). Furthermore, the LGM allows modeling individual or group differences in change trajectories (initial status and change functions) and measuring change at the level of true scores (at the level of latent or manifest variables; Lance et al., 2000). The LGM allows researchers to model change in several focal variables simultaneously. It tests whether the mean-level change of one variable (e.g., OCB) predicts mean-level change in a second variable (e.g., absenteeism) and whether the mean-level change of a second variable (e.g., absenteeism) predicts mean-level change in the first variable (e.g., OCB; Lance et al., 2000). The LGM procedure (see Hounkpatin et al., 2018) requires that constructs (e.g., OCB and absenteeism) be measured on at least three occasions to define higher latent constructs, initial status, and gradient of the variables. Each step of the LGM approach is outlined and presented in Figures 1 and 2. For each survey wave, the OCB measure was represented by a latent score with three observed indicators, whereas the absenteeism score was specified by one observed indicator.

Furthermore, a latent initial level and latent slope were estimated for the OCB-I and absenteeism (frequency and days). Several paths were subsequently estimated with the LGM: the correlation between initial levels of OCB and absenteeism; the change correlation, specifically the correlation between the OCB slope and the absenteeism slope; the prospective OCB-I-level effect, i.e., the path from the initial level of the OCB-I score to absenteeism slope score; the prospective absenteeism effect, represented by the path from the initial level of absenteeism score (frequency or days) to latent slope OCB-I score; the OCB-I-level slope effect represented by the path from the initial level of the OCB-I score to the OCB-I slope; and absenteeism-level slope represented by the path from the initial level of absenteeism score to the absenteeism slope score. Control variables were included as covariates in both models.

We tested a null model to evaluate the percentage of the overall variance between teams (Bliese & Ployhart, 2002). The average between-team variance for the four waves indicates that 6.2% of the variance in absenteeism frequency, 12% of the variance in absenteeism duration, and 23% of the variance in the OCB-I was due to between-team differences over time. These variances indicate significant variances within the teams.

#### Measurement Invariance

Before testing the hypotheses, we examined the longitudinal invariance of the OCB measures across times 1, 2, 3, and 4. Demonstrating measurement equivalence is a prerequisite to conducting the LGM (Lance et al., 2000). The measurement invariance of the OCB construct across the four waves of data collected was evaluated using a CFA framework, as recommended by Vandenberg and Lance (2000). Two models were evaluated using the AMOS 23 program. The fit of these models was assessed using the chi-square statistic (χ^2^) combined with fit indices: comparative fit index (CFI); Tucker–Lewis Index (TLI); and root mean square error of approximation (RMSEA). Model 1 was used to measure configural invariance and contained the items and latent factors for each measurement occasion. The items were specified as loading only on the factor of the corresponding wave. Model 1 showed good fit (χ^2^ = 25.3, *df* = 8, *p* ˂ .01, CFI = 1.00, TLI =.99, RMSEA = .02). Metric invariance was then evaluated with Model 3, which constrained factor loadings to be invariant across waves. Model 3 also showed good fit with the longitudinal data (χ^2^ = 37.8, *df* = 17, p ˂ .01, CFI = .99, TLI =.9, RMSEA = .01). Furthermore, as argued by Vandenberg and Lance (2000), the non-significance of the chi-square difference (Δ χ^2^= 12.5, Δ*df*= 9, *p* > .05) and the low difference between the fit indices of the free model (2) and the fully constrained model (3) indicate that the measurement structure of the OCB construct is invariant (configural and metric invariance) across the four waves of data collection.

### Results

Table 2 presents the descriptive statistics and inter-correlations among variables for the four surveys and absenteeism records for each year.

**Table 2.** Descriptive Statistics and Correlations Among Study Variables (Team Level, N = 168)*.

Variable	*M*	*SD*	1	2	3	4	5	6	7	8	9	10	11	12	13	14	15	16	17	18
1. OCB-I Time 1	7.20	0.56	—																	
2. OCB-I Time 2	7.30	0.45	.35																	
3. OCB-I Time 3	7.37	0.47	.35	.57																
4. OCB-I Time 4	7.52	0.44	.34	.43	.51															
5. OCB-I Average T1-T4	7.35	0.36	.68	.76	.81	.73														
6. OCB-I Change T1-T4	0.10	0.05	−.83	−.32	−.08	.19	−.35													
7. Absenteeism Duration Time 1	2.68	0.24	−.36	−.14	−.14	−.16	−.26	.28												
8. Absenteeism Duration Time 2	2.77	1.08	−.17	−.22	−.11	−.05	−.18	.19	.49											
9. Absenteeism Duration Time 3	2.87	1.17	−.14	−.19	−.26	−.12	−.23	.09	.56	.61										
10. Absenteeism Duration Time 4	2.93	1.32	−.09	−.24	−.27	−.26	−.28	−.02	.46	.50	.71									
11. Absenteeism Duration Average T1-T4	2.82	0.98	−.23	−.24	−.24	−.17	−.29	.16	.74	.78	.89	.81								
12. Absenteeism Duration Change T1-T4	0.08	0.19	.18	−.15	−.21	−.15	−.10	−.25	−.25	.10	.44	.72	.33							
13. Absenteeism Frequency Time 1	2.40	1.06	−.32	−.12	−.10	−.10	−.21	.27	.93	.46	.54	.42	.69	−.23						
14. Absenteeism Frequency Time 2	2.50	0.98	−.10	−.18	−.10	−.04	−.14	.12	.46	.91	.63	.49	.74	.14	.50					
15. Absenteeism Frequency Time 3	2.54	1.02	−.14	−.18	−.24	−.08	−.21	.11	.53	.60	.93	.64	.83	.38	.58	.69				
16. Absenteeism Frequency Time 4	2.65	1.13	−.07	−.23	−.27	−.19	−.25	−.01	.44	.48	.65	.94	.75	.66	.45	.52	.65			
17. Absenteeism Frequency Average T1-T2	2.53	0.84	−.18	−.22	−.22	−.12	−.25	.14	.70	.75	.86	.76	.94	.31	.74	.82	.90	.78		
18. Absenteeism Frequency Change T1-T4	0.08	0.14	.16	−.14	−.24	−.13	−.12	−.22	−.23	.11	.37	.68	.30	.92	−.27	.12	.35	.72	.29	—

*Note.* OCB = organizational citizenship behavior.

* Correlations stronger than .10 or −.10 are significant (*p* < .01).

#### Changes in OCB-I on Changes in Absenteeism

Parameter estimates for the model are presented in Figures 1 and 2. Results in Figure 1 indicate that changes in team-level OCB-I significantly and negatively predict changes in time lost due to absenteeism (β = −.63, *p* < .01), while Figure 2 indicates that changes in team-level OCB-I predict negatively changes in absenteeism frequency (β = −.55, *p* < .01). These negative results support H1.

#### Reverse Relationship: Changes in Absenteeism on Change in OCB-I

H2 proposed that changes in duration and frequency of absenteeism lead to subsequent changes in team-level OCB-I. Results in Figure 1 indicate that changes in time lost due to the absence (days) led to the negative changes OCB-I in teams (days lost: β = −.37, *p* < .05). Moreover, changes in absenteeism frequency (see Figure 2) were negatively associated to changes in OCB-I (frequency β = −.77, *p* < .01), over and above the effect of the initial level of OCB-I, the initial level of absenteeism measure, and the influence of demographic variables and job satisfaction. These findings indicate that a growing absenteeism in teams impede OCB-I, and that OCB-I and absenteeism exert a reciprocal effect on each other.

## Discussion

The focus of the study was on the within-team reciprocal relationship between OCB-I and absenteeism. Building on four repeated measures of multisource data of 5,000 team members in 168 workgroups in large public organization, we found a significant negative relationship between the OCB-I and the absenteeism, viewed as a mildly deviant behavior (Johns & Miraglia, 2015). An increase in team-level OCB-I in a given period is associated to a significant decrease in absenteeism in the same occasion, above and beyond the effect of job satisfaction and initial OCB-I. Testing the inverse relationship, we found that a higher absenteeism is associated with a significant decrease in OCB-I in teams.

### Theoretical Implications

This study makes some important contributions to the OCB and absenteeism literature, as well as to group behavior theories. First and foremost, our study enhances the ongoing discussion on the relationship between OCB and CWB, as explored in previous research (e.g., Dalal et al., 2009; Fox et al., 2012; Griep et al., 2021; Klotz & Bolino, 2013; Spector et al., 2010; Yam et al., 2017). Specifically, we contribute to this literature by showing that higher levels of helping behavior is associated with lower team absenteeism in public sector. This finding contradicts the argument that an increase in OCB-I could lead to more absenteeism due to the fatigue resulting from helping others (Bolino et al., 2015). The negative relationship observed between the increase in OCB-I and absenteeism is good new considering that studies showed that public sector employees engage more in this type of behavior than those in the private sector.

Hence, the current study offers a noteworthy contribution to behavioral theories. First, it stands out as a distinctive addition to the research landscape utilizing the FTNC, which has traditionally focused on examining the impact of social norms (e.g., descriptive and injunctive) on a single behavior, whether it be OCB or CWB (Ehrhart & Naumann, 2004; Jacobson et al., 2015, 2020). There have been limited studies that have both theorized and empirically examined whether changes in social norms pertaining to a specific team behavior can influence changes in social norms regarding other team behaviors. Our research establishes that teams not only anticipate their members’ compliance with prescribed or normative expectations but also their willingness to embrace various compatible behaviors. Normative theories provide a holistic comprehension of the factors influencing behavior. It is crucial to acknowledge that we have not explicitly tested these norms for each individual team, whether through objective or subjective means, owing to the inherent characteristics of our research design. The examination of the intricate relationship between OCB and absenteeism norms in shaping behaviors may represent an avenue for methodological refinement in future studies.

Based on the theoretical framework mobilized, we have posited that when OCB-I levels exceed the norm, team members are more likely to develop and internalize an enhanced sense of citizenship identity. This, in turn, fosters collective motivation to be more consistently present at work. In addition, it is plausible that this alignment in behaviors arises from internal pressures to showcase one’s citizenship identity to avoid potential personal consequences associated with taking more days off than the group typically accepts (Harrison et al., 2006; Ten Brummelhuis et al., 2016). We contend that even in cases of illness, such pressures can lead individuals to show commitment to their citizenship identity to their peers.

Another important contribution of the present study is to have provided preliminary evidence that absenteeism is associated with lower OCB-I. Public teams that exhibit higher absenteeism scores (measured by the number of days and frequency) also tend to show a decrease in their OCB-I levels. Drawing SIT theory, we have argued that higher team absenteeism than normally may elicit a specific collective identity, and a decrease in OCB-I. As absenteeism becomes increasingly pervasive within a group, individuals who are frequently absent may develop a shared identity. Employees with similar absenteeism patterns may unintentionally form an “in-group” within their team or larger organization. They may share common experiences related to absenteeism, such as reasons for taking time off, workplace policies, or the impact of absenteeism on their work and well-being. This shared context can contribute to the development of a shared identity and to reduce helping behavior into two different ways. First, absences limit the opportunities for individuals to offer help or assistance to others. The greater the number of absences on a given day, the lower the likelihood that those present will receive support from their absent coworkers. Second, the formation of in-groups, such as those who are frequently absent versus those with rare absences, can strengthen social categorization and identification with in-group members, potentially resulting in bias in favor of helping those within the same attendance group. This may lead to a higher willingness to assist in-group members with high attendance while showing less inclination to help those perceived as different, namely, low helping between high- and low-attendance group members. Building upon resource allocation theory, we have further posited that elevated team absenteeism, beyond the norm, can result in increased job demands and heightened coordination challenges, consequently limiting the time and energy available for OCB-I within teams. When a higher proportion of team members is absent every day, job overload increases, decreasing the energetic resources available to help others. Team members are thus constrained to make a trade-off between task performance and extra role behavior. The public organization in this study uses a team outcome-based reward system. Therefore, allocating more time to task performance rather than OCB is probably viewed as a wiser time allocation strategy (Bergeron, 2007; Bergeron et al., 2014).

A noteworthy finding is that changes in absence frequency were more strongly negatively related to OCB-I changes than changes in time lost due to absences (−.77 vs. −.37). This finding is more likely to contribute to discussions on the significance of employing both absenteeism indicators (Johns & Al Hajj, 2016). It is plausible that within a team, recurrent 1-day absences have a more pronounced dampening effect on helping behavior compared with a single 5-day absence. Several factors may elucidate this phenomenon. First, a 5-day absence permits a more efficient allocation of resources and efforts, along with the establishment of effective preventive backup strategies, thus leading to reduced work burden. Second, 5-day absences are typically more predictable compared with frequent, unplanned 1-day absences.

### Practical Implications

The results of this study suggest some practice guidance and implications for public managers and human resource departments. First, decision-makers should regularly monitor, not only to magnitude of OCB and absenteeism within public service units but also the direction of their changes (increase or decrease), as well as to why and when both behaviors are connected. Consequently, they can provide appropriate solutions before dysfunctionalities, such as excessive absenteeism and disengagement in various helping behaviors become a cultural norm. A specific attention must be paid to the various trends that allow managers to identify the units experiencing a positive trend to avoid introducing binding control mechanisms, which are unwanted within units with a positive OCB-I trend and with a decreased absenteeism pattern.

Second, while increasing helpful behaviors in teams can help to prevent an absenteeism escalation, this type of behavior should be encouraged by using various human resource management practices (Halbesleben & Wheeler, 2015; Tremblay, 2019; Tremblay & Simard, 2018). First, public organizations should communicate clear expectations for OCB-I as part of their values and culture. Second, team leaders should lead by example by performing pro-social behaviors and prioritize interpersonal relationships to inspire employees to engage in OCB-I. Training programs that focus on interpersonal skills and teamwork should be offered to help employees develop the capabilities to engage in OCB-I. Implementing rewards and recognition for their extra miles in assisting colleagues is more likely to motivate pubic employees to continue engaging in such behaviors.

However, managers must be aware that the efforts and resources dedicated to promoting helping behaviors in their teams may not help them reach their goals if themselves or the organization accepts excessive absenteeism and increasing lower presence at work. An increasing absenteeism trend, and principally absenteeism frequency, risk to encourage team members to focus exclusively on required and urgent tasks and duties, which in turn may affect negatively some important public performance indicators (N. P. Podsakoff et al., 2009, 2014). Reducing abnormal absenteeism in the public sector requires a combination of policies, practices, and a supportive environment. First, public decision-markers should develop and communicate clear attendance policies including detail reporting absences and consequences for excessive absenteeism for employees. Public employees need to know what is considered acceptable and what isn’t. Hold orientation sessions for new hires and provide periodic reminders and updates to the entire staff should be considered. In the same vein, public authorities should provide prescriptive information about the consequences of increasing and non-planned use days off for the organization (e.g., the cost for the public organizations, impact on budgets), the teams (e.g., time and quality of service delivery), and individuals (e.g., work overload, higher stress). Second, an absenteeism policy should include a clear progressive discipline process detailing a progressive approach for excessive absenteeism. Third, considering to implement incentive programs that reward good attendance as bonuses or recognition. A recognition program may include to publicly acknowledge and celebrate employees with outstanding attendance. This can include awards, certificates, or public praise during team meetings or through newsletters.

We estimated the practical significance of the impact of OCB-I changes on days of absenteeism in the public organization studied. According to our estimates, public teams with a positive OCB-I change score of 1 *SD* below the mean exhibited an average of 3.92 days of absenteeism. In contrast, teams with an OCB-I change score of 1 *SD* above the mean had 2.10 days of absenteeism. For an organization with 15,000 employees and an estimate that each day of absenteeism costs the employer US$500.00 (Hausknecht et al., 2008), these additional 1.82 days represent a potential annual loss of US$13,650,000. Although eliminating absenteeism is unrealistic (Hausknecht et al., 2008), preventing an excessive increase in such a withdrawal behavior would yield a substantial payoff.

### Research Contributions, Strengths, Limitations, and Future Research

In addition to its theoretical contribution, this study has several methodological strengths. These include the sample large size comprising more than 5,000 respondents in 168 workgroups in large public utility organization and the longitudinal research design made up of four measurement times for each variable assessed (OCB-I and two absenteeism indices). Moreover, absenteeism measures were provided by an external source, reducing the bias normally associated with common variance and social desirability (O’Brien & Allen, 2008). Another strength is that this research has tested the reciprocal links between the variables assessed and examined their interplay over a longer period than previous studies (Methot et al., 2017).

Nevertheless, this study has several notable limitations. First, the survey was carried out in only one public organization, reducing its external validity and generalizability to other public contexts. However, the assessment of a single organization has the advantage of allowing for efficient control of several endogenous variables that may affect the relationships (e.g., absenteeism policy, working conditions, and human resources management practices). Second, the study relied on self-report measures to evaluate OCB-I, hence may suffer from a systematic inflation bias. Common variance bias was reduced because OCB-I and absenteeism were separated by a time lag, and that aggregated individual perceptions were more likely to reduce error by averaging out random individual-level errors and biases (Robinson & O’Leary-Kelly, 1998). One meta-analysis (Carpenter et al., 2014) suggests that self-rated OCB must be considered a viable and valid method of measuring the OCB. Third, even though withdrawal behaviors are considered an important CWB dimension (Gruys & Sackett, 2003), whether objective voluntary absenteeism is perceived as harmful to employees, to the organization or to unions is debatable. Fourth, as point out previously, we have not explicitly undertaken testing the change in absenteeism and OCB-I norms within each team. By neglecting to explicitly test these normative developments, we miss the opportunity to fully understand how changes in absenteeism and OCB norms can influence team dynamics. A thorough examination of these normative changes at the team level could provide more precise insights and constitute an area for further exploration in subsequent investigations. Another limitation of the study concerns not assessing the mechanisms linking OCB and absenteeism (e.g., citizenship identity, presenteeism, workload, social norms, energy). However, we controlled for various job satisfaction dimensions for an alternative explanation.

This study points to several avenues for further research. First, previous studies revealed that the relationship between the OCB and outcome variables at the team level varied across contextual variables such as job characteristics (Nielsen et al., 2012), organizational structure (DeGroot & Brownlee, 2006), and employment rate (Sun et al., 2007). This study raises interesting research questions for: (a) To what extent do task, outcome, and reward interdependence affect the relationship between OCB and absenteeism? (b) To what extent is the relationship between OCB and CWB is less strongly affected by flexible than bureaucratic structures? (c) Why are OCB and absenteeism mutually related? Possible mediating processes include citizenship fatigue, job load, and feeling of energy (Bolino et al., 2015; Lam et al., 2016; Ybema et al., 2010). A future research is also needed to know if the social normative norms (descriptive vs. injunctive) exert a similar effect in OCB-CWB versus CWB-OCB relationships. Finally, a natural extension of this study is to explore targets other than co-workers, and to examine whether group OCB targeted toward the supervisor, organization, and service users or citizens may have differential effects on team absenteeism.

## Conclusion

This study highlights how public teams dynamically use OCB-I and absenteeism to respond to a workplace context. We found that when team OCB-I increases, absenteeism decreases, and when absenteeism increases, OCB-I decreases. Furthermore, absence frequency affects more negatively OCB-I changes than time lost due to absences, suggesting that repeated 1-day absences in a team weaken helping behavior more than a 5-day absence. This study contributes to public management science, and more specifically to team dynamics, by showing the relevance of pursuing within-team research because relationships at this level are not always similar or isomorphic to those at between-level or at within-person level.
